# Changes in Expression Level of *OsHKT1;5* Alters Activity of Membrane Transporters Involved in K^+^ and Ca^2+^ Acquisition and Homeostasis in Salinized Rice Roots

**DOI:** 10.3390/ijms21144882

**Published:** 2020-07-10

**Authors:** Mohammad Alnayef, Celymar Solis, Lana Shabala, Takaaki Ogura, Zhonghua Chen, Jayakumar Bose, Frans J. M. Maathuis, Gayatri Venkataraman, Keitaro Tanoi, Min Yu, Meixue Zhou, Tomoaki Horie, Sergey Shabala

**Affiliations:** 1 Tasmanian Institute of Agriculture, University of Tasmania, Hobart, TAS 7005, Australia; mohammad.alnayef@utas.edu.au (M.A.); C.Solis@westernsydney.edu.au (C.S.); L.Shabala@utas.edu.au (L.S.); togura@g.ecc.u-tokyo.ac.jp (T.O.); jayakumar.bose@adelaide.edu.au (J.B.); meixue.zhou@utas.edu.au (M.Z.); 2 School of Science and Health, Western Sydney University, Penrith, NSW 2751, Australia; Z.Chen@westernsydney.edu.au; 3 International Research Centre for Environmental Membrane Biology, Foshan University, Foshan 528000, China; yumin0820@hotmail.com; 4 Graduate School of Agricultural and Life Sciences, University of Tokyo, Tokyo 113-8657, Japan; uktanoi@g.ecc.u-tokyo.ac.jp; 5 Hawkesbury Institute for the Environment, Western Sydney University, Penrith, NSW 2751, Australia; 6 Australian Research Council Centre of Excellence in Plant Energy Biology, School of Agriculture, Food and Wine, University of Adelaide, Glen Osmond, SA 5064, Australia; 7 Department of Biology, University of York, York, YO10 5DD, UK; frans.maathuis@york.ac.uk; 8 Plant Molecular Biology Laboratory, M.S. Swaminathan Research Foundation, Chennai 600113, India; gayatri@mssrf.res.in; 9 Division of Applied Biology, Faculty of Textile Science and Technology, Shinshu University, Nagano 386-8567, Japan; horie@shinshu-u.ac.jp

**Keywords:** salinity stress, xylem loading, sodium, potassium, SKC1, HAK5, GORK, RBOHD, epidermis, stele

## Abstract

In rice, the *OsHKT1;5* gene has been reported to be a critical determinant of salt tolerance. This gene is harbored by the *SKC1* locus, and its role was attributed to Na^+^ unloading from the xylem. No direct evidence, however, was provided in previous studies. Also, the reported function of *SKC1* on the loading and delivery of K^+^ to the shoot remains to be explained. In this work, we used an electrophysiological approach to compare the kinetics of Na^+^ uptake by root xylem parenchyma cells using wild type (WT) and NIL(*SKC1*) plants. Our data showed that Na^+^ reabsorption was observed in WT, but not NIL(*SKC1*) plants, thus questioning the functional role of *HKT1*;*5* as a transporter operating in the direct Na^+^ removal from the xylem. Instead, changes in the expression level of *HKT1*;*5* altered the activity of membrane transporters involved in K^+^ and Ca^2+^ acquisition and homeostasis in the rice epidermis and stele, explaining the observed phenotype. We conclude that the role of *HKT1*;*5* in plant salinity tolerance cannot be attributed to merely reducing Na^+^ concentration in the xylem sap but triggers a complex feedback regulation of activities of other transporters involved in the maintenance of plant ionic homeostasis and signaling under stress conditions.

## 1. Introduction

The exposure of plants to high levels of salt in the rhizosphere results in the accumulation of toxic levels of Na^+^ and Cl^-^ in the cellular and extracellular compartments affecting cellular metabolic activity and, thus, overall plant performance. Salt-tolerant plants have developed many mechanisms for minimizing radial and long-distant transport of Na^+^ and preventing its accumulation in metabolically active tissues. These mechanisms, known as salt exclusion mechanisms, include: (1) minimizing Na^+^ entry by the selective uptake of ions by root cells; (2) Na^+^ exclusion from the root back to rhizosphere; (3) preventing xylem Na^+^ loading; (4) enhanced retrieval of Na^+^ from xylem vessels; and (5) the control of phloem ion loading [1,2,3,4,5].

Glycophytic plants, such as rice, use these salt-excluding mechanisms as their primary strategy for preventing the toxic accumulation of salt in photosynthetic tissues [6]. In such plants, maintenance of a relatively high K^+^/Na^+^ ratio, especially in shoots, is a key factor in the development of salt tolerance [7,8,9]. The concerted activity of a large number of transporters located in the plasma and vacuolar membranes plays an important role in determining the K^+^/Na^+^ ratio in plant cells, through Na^+^, K^+^-selective and non-selective pathways [8,10,11,12]. The long-distance transport of Na^+^ in plants is determined by the rate of *net* xylem loading. The latter is a result of an orchestrated operation of transporters mediating Na^+^ release into the xylem (e.g., via salt overly sensitive 1 (SOS1) or chloride-dation cotransporter (CCC) transporters or non-selective cation channels; [2,13]) and transporters involved in the retrieval of Na^+^ from the transpiration stream. Key players in the latter process are HKT1 transporters.

HKT1 transporters (subfamily I) belong to the TrK/Ktr/HKT family (Transporter of K^+^/K^+^ transporter/High-affinity K^+^ Transporter) that is implicated in various functions, from K^+^ or Na^+^ uptake to the maintenance of membrane potential, ion homeostasis, and Na^+^ recirculation from the shoot to the root [14]. The HKT family can be divided into two distinct subfamilies based on their ion selectivity [15,16]. The transporters in subfamily I, such as *OsHKT1;1* and *OsHKT1;5*, are selective for Na^+^, while those in subfamily II are selective for either Na^+^ or K^+^ or, alternatively, transport both ions [16,17,18]. The *Arabidopsis* genome encodes only one gene, *AtHKT1;1*, that was demonstrated to function in Na^+^ transport and exclusion from leaves [19,20,21]. In durum wheat, the *TmHKT1*;*5-A* gene was shown to be present in the *Nax2* QTL that contributes to lowering Na^+^ levels in leaves [22,23], and the near-isogenic line (NIL) of durum wheat harboring *TmHKT1*;*5-A* increased grain yield by 25% under saline conditions [24]. It was also suggested that *TaHKT1*;*5-D* is the candidate gene for the *Kna1* locus in bread wheat, which has long been known as essential for leaf Na^+^ exclusion and the maintenance of a high K^+^/Na^+^ ratio in leaves [25,26]. Another important allele for Na^+^ exclusion, *Nax1*, was identified in wheat by QTL analysis; in contrast to *Nax2*, this locus was able to confer Na^+^ unloading not only in roots but also in leaf sheaths [22]. *Nax1* was later predicted to be the *TmHKT1*;4-A2 gene which is also the class I HKT HKTs [27]. However, more recent studies suggested that the above *Nax* loci harboring *HKT1*;*4* or *HKT1*;*5* genes involved in the retrieval of Na^+^ back into the root stele but also plays a role in reducing the rate of Na^+^ loading into the xylem by modulating activity and expression levels of the *SOS1* gene [28]. Thus, the actual role of *HKT1* in Na^+^ unloading from the xylem may be not as straightforward as previously believed.

In the rice genome, a total of nine HKT genes have been found, divided between subfamilies I and II [17,29,30]. One of them, *OsHKT1*;*5*, has been narrowed down as a determinant of salt tolerance by QTL analysis and attributed to the *SKC1* locus [31]. These authors showed that near-isogenic (NIL) *SKC1* plants accumulated less Na^+^ and had a more salt-tolerant phenotype. Based on GUS (β-glucuronidase) staining (*SKC1* promoter), Na^+^ analysis of the xylem sap, and transport assays in *Xenopus* oocytes, the authors concluded that the *SKC1* locus encoded an Na^+^-selective *HKT1*;*5* transporter that was expressed in the xylem parenchyma and was involved in the unloading of Na^+^ from the xylem. However, no direct evidence for the operation of *HKT1*;*5* in the xylem parenchyma was provided. Also, the reported xylem sap data was collected from plants treated with rather mild (25 mM NaCl) salinities while all the genetic data in the present work refer to much more severe (140 mM NaCl) treatment.

Ren et al. [31] also reported a major difference in loading and delivery of K^+^ to the shoot between *SKC1* and WT lines. In fact, the name of the locus *per se* (*SKC1*) stands for “shoot K^+^ content 1” and implies that this locus has a major impact on plant K^+^ homeostasis. This is further echoed by the fact that in wheat, both *Nax1* and *Nax2* loci have also been shown to increase the rate of K^+^ transport from root to shoot [22]. Also, the *SKC1* locus was later found to be localized within the *Saltol* locus, a major salt tolerance QTL in rice [32], and the latter was also shown to be an important determinant of K^+^ homeostasis during salt stress [33]. A role for *OsHKT1;5* in mediating Na^+^ exclusion in the phloem has also been shown [34]. Taken together, these results question the causal link between the observed salt-tolerant phenotype and the role of *HKT1*;*5* in Na^+^ retrieval from the xylem and point at the possibility of operation of some other (complementary) mechanisms. Surprisingly, this issue has not been properly addressed in direct functional experiments. In their work, Ren et al. [31] explained elevated K^+^ content in NIL *SKC1* rice plants as coming from some “*interplay between individual HKT members and other transporters*” suggesting that “*one ion can affect the transport and accumulation of another*”. No further details have been elucidated, and, to the best of our knowledge, no follow-up work has been conducted to address this issue. Filling this void in our knowledge is the main aim of this study.

## 2. Results

**Figure 1:**
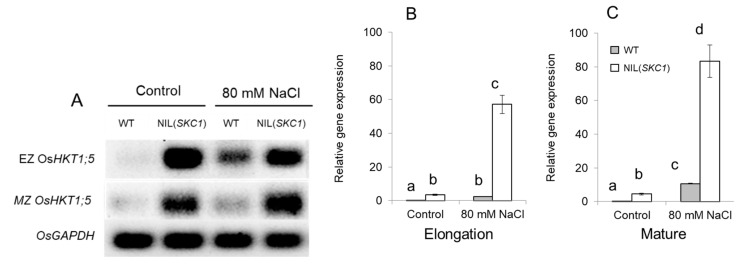
(**A**) qRT-PCR analysis of expression of *OsHKT1;5* in the elongation (EZ) and mature (MZ) root zones under control and salinity (80mM NaCl, seven days) conditions. (**B**,**C**) Relative gene expression values normalized by the corresponding amount of *OsGAPDH* as an internal control. Mean ± SE (*n* = 3 technical each consisting of six pooled individual plants). The numbers labeled by different letters are statistically different at *p* < 0.05.

In our work, we compared the responses of wild type (WT; cv. Koshihikari) and its near-isogenic line with a short segment containing *SKC1* [abbreviated here as NIL(*SKC1*)] rice plants to salinity treatment. In our hands, NIL(*SKC1*) plants showed significantly higher expression of *HKT1*;*5* in both the elongation and mature root zones compared with WT (Figure 1A), and *HKT1*;*5* transcript levels were significantly upregulated by salinity (Figure 1B,C). Thus, at the operational level, NIL(*SKC1*) plants could be deemed as an *HKT1*;*5* overexpressing line.

**Figure 2:**
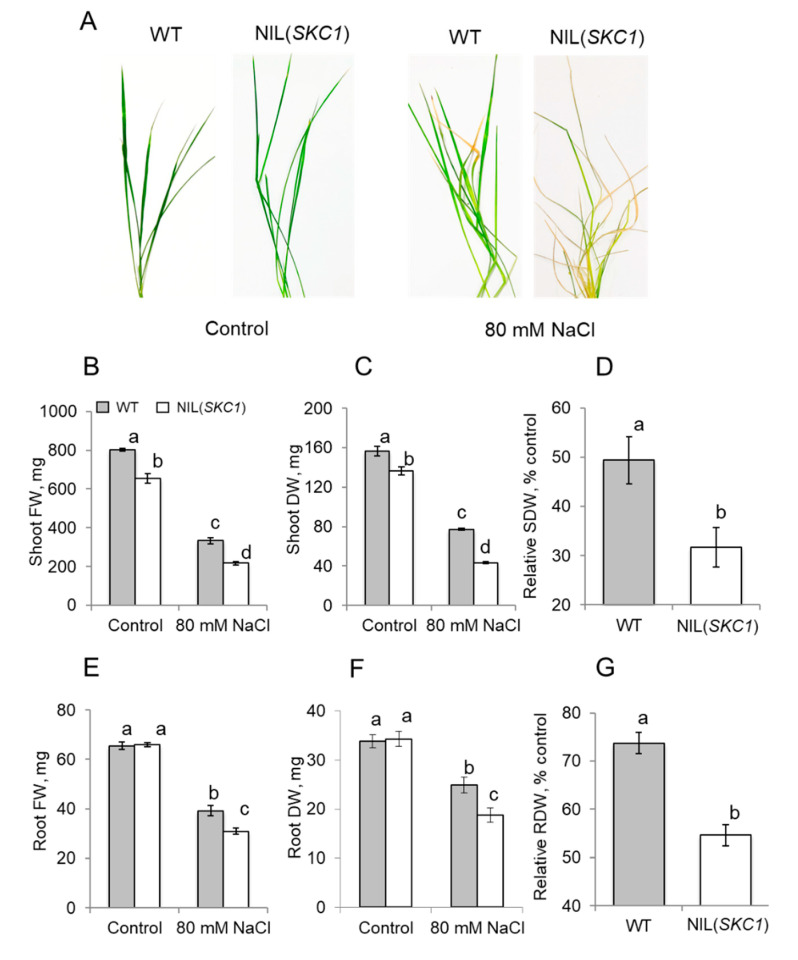
Morphological changes of wild-type (WT) and SKC1-NIL plants grown under control and saline (80 mM NaCl for seven days) conditions. (**A**) plant phenotype; (**B**–**D**) changes in shoot weight; (**E**–**G**) changes in root weight. FW, fresh weight; DW, dry weight. SDW, shoot dry weight; RDW, root dry weight. Mean ± SE (*n* = 5). The numbers labeled by different letters are statistically different at *p* < 0.05.

Root growth in NIL(*SKC1*) plants were similar to those in WT (Figure 2E,F). In contrast, the shoot fresh (FW) and dry (DW) weights were smaller in *SKC1* plants grown under control conditions (Figure 2B,C). Salinity stress resulted in a significant (*p* < 0.05) reduction in both root and shoot growth (Figure 2). In contrast to Ren et al. [31], we used less severe treatment (80 mM for one week compared to their 140 mM for 10 days). Under these conditions, the NIL(*SKC1*) line showed a more sensitive phenotype, with a larger proportion of chlorotic and necrotic leaves (Figure 2A) and significantly lower relative shoot (Figure 2D) and root (Figure 2G) dry weights compared to WT.

**Figure 3:**
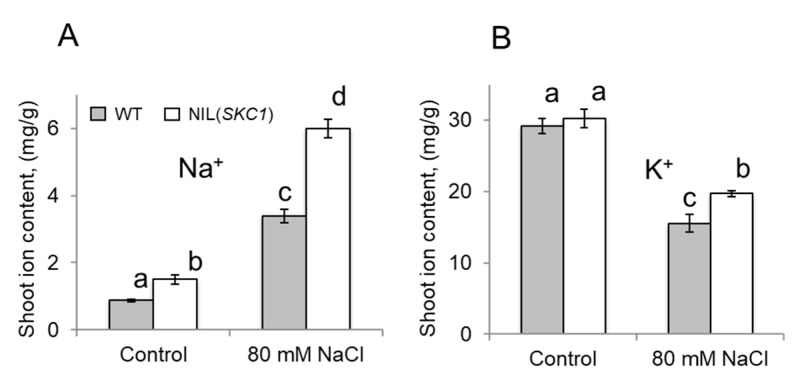
Shoot Na^+^ (**A**) and K^+^ (**B**) content of WT and NIL(*SKC1*) plants grown under control and saline (80 mM NaCl for seven days) conditions. Mean ± SE (*n* = 5). Numbers labelled by different letters are statistically different at *p* < 0.05.

Similar to Ren et al. [31], NIL(*SKC1*) plants also accumulated more K^+^ under saline conditions (Figure 3B) but, interestingly, also had more Na^+^ (Figure 3A). The latter result is clearly inconsistent with the notion that *SKC1* operates in Na^+^ removal from the shoot.

The conclusion made by Ren et al. [31] about HKT1;5 operating in Na^+^ retrieval from the xylem into the parenchyma cells was based on the detection of GUS activity mainly in the parenchyma cells bordering the xylem vessels. However, no direct evidence for *in planta* HKT1;5 operation was given. To address this issue, we have employed the microelectrode ion flux estimation (MIFE) technique to directly measure fluxes of Na^+^ at the xylem/parenchyma interface of rice NIL(*SKC1*) lines.

In addition to comparing WT with NIL(*SKC1*) (operating as *HKT1*;*5* overexpressing line at the functional level), we have also included *O. sativa* cv. Dongjin 4A-02764 mutant allele genotype harboring a T-DNA insertion in *OsHKT1*;*5* used in Kobayashi et al. [34]. These plants showed a reduction in *OsHKT1*;*5* expression in roots in comparison to the level in WT plants (as evidenced from the results of the RT-PCR using 10-day-old whole rice seedlings; see Figure 7A and Supplementary Figure S2 from [34]) and, therefore, at the functional level could be termed as a knock-down (KD). As this allele has the *japonica* rice cv. Dongjin background, fluxes were normalized for the appropriate WT, to make results comparable.

**Figure 4:**
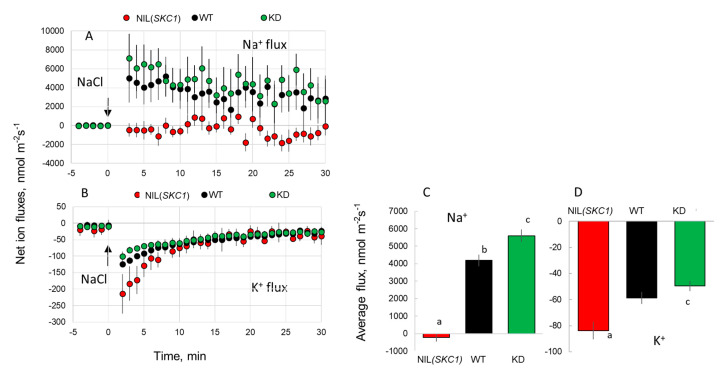
Net Na^+^ and K^+^ fluxes measured from the xylem parenchyma cells in response to acute 80 mM NaCl treatment. (**A**,**B**) transient Na^+^ (**A**) and K^+^ (**B**) fluxes measured from NIL(*SKC1*), WT, and *Oshkt1;5* (4A-02764) knock-down lines (KD). As the latter allele has a japonica rice cv. Dongjin background, fluxes were normalized for the appropriate WT, to make the results comparable with those measured from NIL(*SKC1*) and its wild type. (**C**,**D**) mean Na^+^ and K^+^ fluxes over the 30 min period after stress onset, respectively. Mean ± SE (*n* = 4 to 9). For all MIFE measurements, the sign convention is “influx positive”. The numbers labeled by different letters are statistically different at *p* < 0.05.

When 80 mM NaCl was applied to the root stele of WT plants (mimicking an increase in the xylem sap Na^+^ concentration), strong and sustained net Na^+^ uptake was measurable (Figure 4A,C). In functional terms, this uptake would be consistent with reabsorption of Na^+^ from the xylem in intact roots (either via *HKT1*;*5* or by some other transport system). However, in the NIL(*SKC1*), such uptake was absent. On the contrary, the KD line showed even slightly higher net Na^+^ uptake by the xylem parenchyma cells than the WT (Figure 4A,C). These results are consistent with the above whole-plant Na^+^ content data shown in Figure 2.

We then measured the effects of NaCl on K^+^ transport across the plasma membrane of root stellar tissues (Figure 4B,D). In all three lines, the addition of NaCl to the bath solution resulted in a transient net K^+^ efflux. In functional terms, *in planta*, this would be equivalent to NaCl-induced K^+^ loading into the transpiration stream. The magnitude of K^+^ efflux was NIL(*SKC1*) > WT > KD and consistent with our data (Figure 3B) and that from Ren et al. of higher K^+^ accumulation in the shoot of the NIL(*SKC1*) line.

**Figure 5:**
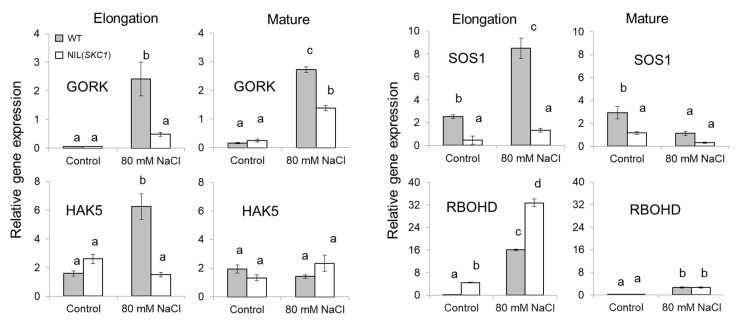
Effect of salinity on the expression of *GORK*, *HAK5*, *SOS1*, and *RBOHD* genes in NIL(*SKC1*) line and its wild type. Mean ± SE (*n* = 3 technical samples each consisting of 6 pooled individual plants). The numbers labeled by different letters are statistically different at *p* < 0.05.

Ion homeostasis in plants is regulated by numerous positive and negative feedback loops, and changes in the expression level/activity of one transporter may affect the functionality of others. Accordingly, we compared changes in the expression levels of some key genes affecting plant ion homeostasis between roots of WT and *SKC1* plants. In addition to genes controlling K^+^ and Na^+^ homeostasis (e.g., *SOS1* for Na^+^/H^+^ exchange; *GORK* channel for K^+^ efflux; *HAK5* for high affinity K^+^ uptake), we also looked at the changes in the expression levels of *RBOHD*, a gene that encodes an NADPH (nicotinamide adenine dinucleotide phosphate) oxidase and thus, mediates stress-induced Ca^2+^ signaling in plants by controlling operation of the “ROS-Ca^2+^ hub” at the plasma membrane [35]). A significant difference was found in the expression levels of many transporter genes (Figure 5); this difference also showed strong time- and tissue-dependence (e.g., different response patterns in the elongation and mature root zones). Specifically, NIL(*SKC1*) plants had reduced expression of *RBOH* transcripts under both control and saline conditions (in both root zones; Figure 5) but much higher expression of *RBOHD* in the elongation zone. Also, when exposed to salinity, the NIL(*SKC1*) line showed reduced *GORK* expression but increased expression of *RBOHD* transcripts compared with WT (Figure 5). Also, there were lower SOS1 transcript levels in both root zones.

**Figure 6:**
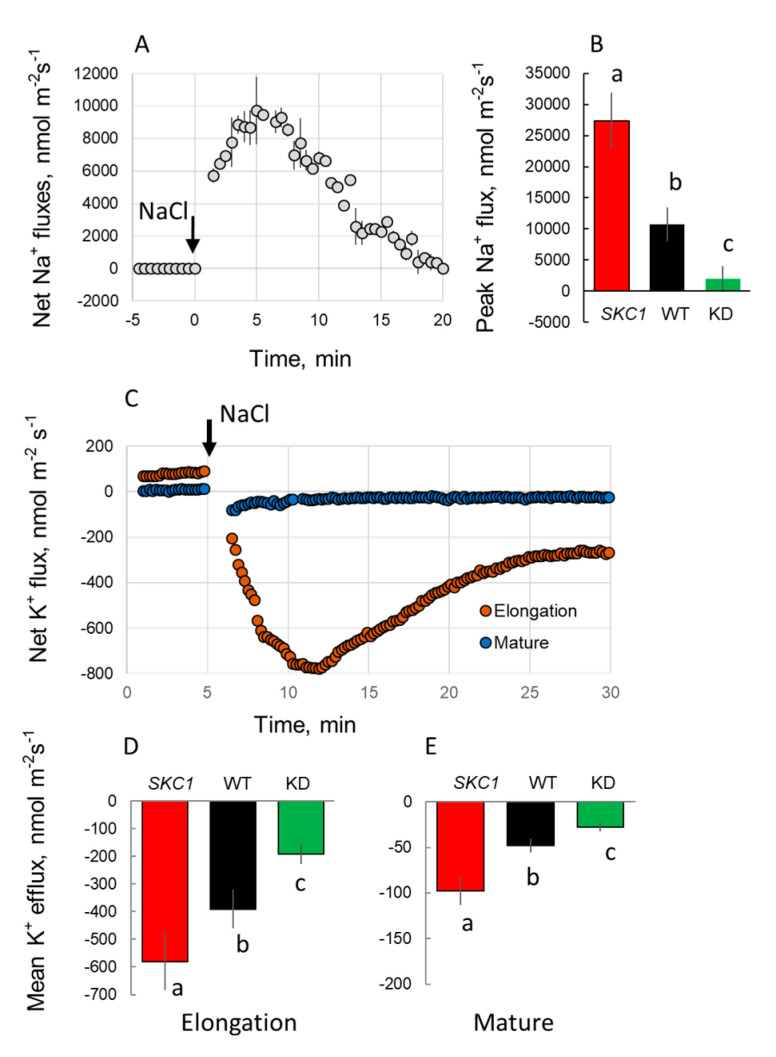
Net Na^+^ and K^+^ fluxes measured from the epidermal root cells in response to acute 80 mM NaCl treatment in functionally different root zones. (**A**) transient Na^+^ fluxes measured from the elongation zone of WT plants. (**B**) the magnitude (peak Na^+^ uptake value) measured from NIL(*SKC1*), WT and *Oshkt1;5* (4A-02764) knock-down (KD) lines in the elongation zone. Measurements from the mature zone have not been conducted as SOS1 genes mediating Na^+^ extrusion are expressed predominantly in the root apex [37]. (**C**) transient K^+^ fluxes measured from the elongation and mature zones of WT plants. (**D**,**E**) mean K^+^ fluxes measured over the 30 min period after stress onset from elongation and mature root zones of NIL(*SKC1*), WT, and *Oshkt1;5* plants, respectively. Mean ± SE (*n* = 4–9). The numbers labeled by different letters are statistically different at *p* < 0.05.

We then asked if the altered *HKT1*;*5* expression could affect the functional activity of membrane transporters in the root epidermis. For doing this, we first compared the patterns of Na^+^ uptake in root epidermis (Figure 6A,B) in all three lines. This uptake was strongest in NIL(*SKC1*) followed by WT and then by the KD lines indicating that changes in the expression levels of *HKT1*;*5* in the root *stellar* tissue strongly affected Na^+^ uptake ability in the root epidermis. Further, the NaCl-induced K^+^ loss from epidermal root cells was as: NIL(*SKC1*) > WT > KD (Figure 6C–E). This pattern was observed in both root zones. Similar to previous studies on various species (e.g., [8,36]), K^+^ loss from the elongation zone (EZ) was much stronger (about six-fold) compared with the MZ.

As commented above, transcriptional studies showed a significant difference in profiles of *SOS1* and *RBHOD* between WT and NIL(*SKC1*) lines grown under saline conditions (Figure 5).

**Figure 7:**
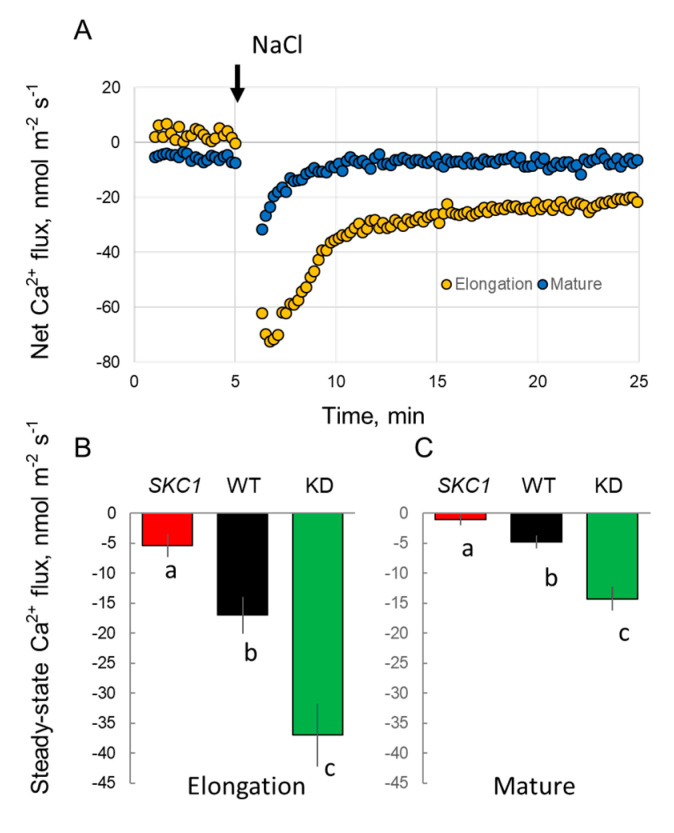
Net Ca^2+^ fluxes measured from the epidermal root cells in response to acute 80 mM NaCl treatment in functionally different root zones. (**A**) transient Ca^2+^ fluxes measured from the elongation and mature zones of WT plants. (**B**,**C**) mean Ca^2+^ fluxes measured over the 30 min period after stress onset from elongation and mature root zones of NIL(*SKC1*), WT and *Oshkt1;5* plants, respectively. Mean ± SE (*n* = 4–9). The numbers labeled by different letters are statistically different at *p* < 0.05.

Th operation of Na^+^/H^+^ exchangers is controlled by the SOS pathway [5]; the essential step in this process is salt stress-induced elevation in the cytosolic free Ca^2+^. Such changes in cytosolic Ca^2+^ are also essential for the regulation of NADPH oxidase operation that impacts the activity of cation channels via apoplastic ROS production [35,38]. Accordingly, we compared patterns of NaCl-induced net Ca^2+^ fluxes in rice *HKT1*;*5* lines used above (Figure 7). Acute NaCl treatment caused transient Ca^2+^ efflux, most likely as a result of the Donnan exchange in the cell wall [39]. However, this response was stronger in EZ, and at the end of the transient response, net Ca^2+^ flux values remained negative, suggesting the involvement of some active Ca^2+^ efflux system. This net Ca^2+^ efflux was changed in a sequence NIL(*SKC1*) < WT < KD suggesting that the overexpression of *HKT1*;*5* in NIL(*SKC1*) had compromised operation of one of the Ca^2+^ efflux systems (either Ca^2+^-ATPase or CAX exchanger; [40]).

**Figure 8:**
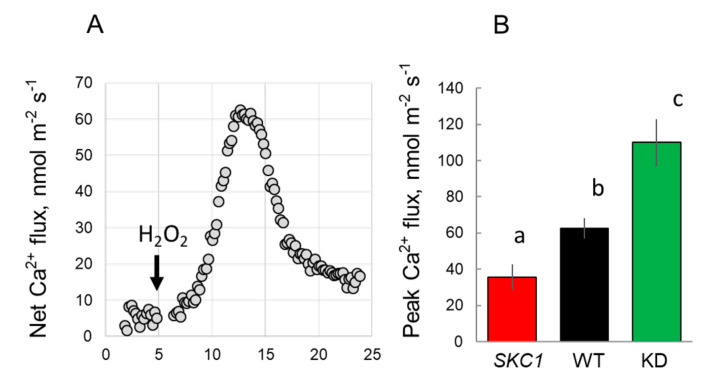
Net Ca^2+^ fluxes measured from the epidermal cells in the elongation region of rice roots in response to 10 mM H_2_O_2_ treatment. (**A**) transient Ca^2+^ fluxes measured the elongation zone of WT plants. (**B**) peak Ca^2+^ flux measured from the epidermal cells of NIL (*SKC1*), WT and *Oshkt1;5* (KD) plants. Mean ± SE (*n* = 4–9). The numbers labeled by different letters are statistically different at *p* < 0.05.

H_2_O_2_ plays an important role in the adaptive responses to the salinity and sensitivity of cation-permeable channels to H_2_O_2_, which often explains tissue- and genotypic-variability in salinity stress tolerance [41,42]. Accordingly, we compared the magnitude of H_2_O_2_-induced Ca^2+^ fluxes in the root epidermis of the three genotypes (Figure 8).

Similar to previous findings, H_2_O_2_ triggered transient Ca^2+^ influx that was KD > WT > NIL(*SKC1*). Thus, overexpressing *HKT1*;*5* in NIL(*SKC1*) had compromised sensitivity of Ca^2+^-permeable cation channels to H_2_O_2_, potentially affecting the ability of the plant to sense and respond to salt stress.

## 3. Discussion

HKT1 transporters have been on researchers’ radar since 1994 [43] and are firmly associated with plant salinity tolerance; this is specifically true for *HKT1*;*5*. However, the causal link between the operation of *HKT1*;*5* and plant phenotype remains complicated. The general consensus is that *HKT1*;*5* transporters are expressed in the xylem parenchyma and operate in the reabsorption of Na^+^ loaded into the xylem [1,24]. However, to the best of our knowledge, no direct measurements of Na^+^ fluxes across the xylem/parenchyma boundary have been reported so far. Here, we used non-invasive Na^+^ selective microelectrodes and compared kinetics of Na^+^ uptake by root parenchyma cells between NIL(*SKC1*) (overexpressing *HKT1*;*5*) and WT plants (Figure 4). In our protocols, adding Na^+^ to the bath solution surrounding the xylem parenchyma tissue would mimic the *in planta* situation when Na^+^ is loaded into the xylem. As shown in Figure 4, Na^+^ reabsorption (net Na^+^ influx) was clearly observed in WT but not in NIL *SKC1* plants. These observations question the functional role of *HKT1*;*5* as a transporter operating in direct Na^+^ removal from the xylem.

Several possible explanations could be considered to explain this apparent conundrum. First, even though the GUS staining is strongest in the xylem parenchyma, *HKT1*;*5* may be potentially also present in the root epidermis). Indeed, while the expression of the *OsHKT1;5* gene in stellar cells is robust, it may be much lower in the epidermis and below the detection limit of the GUS staining analysis. Yet, when overexpressed in epidermal tissue, *HKT1*;*5* could potentially increase the rate of root Na^+^ uptake (and then the subsequent xylem loading). The supporting evidence comes from MIFE net Na^+^ flux data measured from the root epidermis (Figure 6). Here, the strongest Na^+^ uptake by roots was observed in NIL *SKC1* plants followed by WT and then by KD plants. Thus, if *HKT1*;*5* is overexpressed in both xylem parenchyma and root epidermis, the beneficial role of the stele-based *HKT1*;*5* in removing Na^+^ from the xylem may be overridden by a concurrent increase in root Na^+^ uptake in epidermis. This could explain the fact that the overall accumulation of Na^+^ in the shoot was higher in the NIL(*SKC1*) line compared with WT (Figure 3). In a sense, this scenario is analogous to the expression/operation of SOS1 transporters, which are also expressed in both root tissues (Shi et al. 2000). However, the q-PCR analysis on *OsHKT1;5* found in the RiceXPro database indicated that *OsHKT1;5* expression is quite specific in the root stellar cell region of the mature zone (https://ricexpro.dna.affrc.go.jp/), and the same conclusion was derived from the immuno-staining data [34].

It is also plausible to suggest that the *SKC1* locus may also contain some other genes that could attribute to transcriptional, translational and the post-translational changes in *HKT1*;*5* function overall. The colocalization of transcription factors are known to underpin major QTL effects [44], and this could be the case in our study. Finally, plant functioning relies on a myriad of signaling loops, and changes in the transcript levels of one gene might have feedback effects on the operation of others, including those encoding different transporters. In our case, elevated *HKT1*;*5* transcript levels in NIL(*SKC1*) plants had a significant impact on the expression levels of transporters mediating plant K^+^ (GORK, HAK5) and ROS (reactive oxygen species; RBOHD) homeostasis (Figure 5), thus modulating their functional activity (Figure 6 and Figure 7). Recently, El Mahi et al. [45] have shown that *HKT8/HKT1;5/SKC1* transcript level is significantly down-regulated in roots of *sos1* mutant rice plants, and it was suggested that this down-regulation could represent a root-protecting mechanism limiting the back-flow of Na^+^ to roots that are already exposed to high Na^+^ contents. Also, the bypass flow plays a significant role in Na^+^ uptake in rice [46]. In *Arabidopsis*, *RBOHD* controls lignification and suberin deposition in root endodermis [47]. Given that *RBOHD* expression levels were altered in NIL(*SKC1*) plants in our study, alterations in a sodium uptake through apoplast are plausible.

Salinity stress tolerance in plants is associated with the ability of their roots to retain K^+^ when exposed to salinity [9,48]; this is specifically true for rice [36]. In our case, the magnitude of NaCl-induced K^+^ efflux was strongest in NIL (*SKC1*) plants (Figure 6C–E), explaining its salt-sensitive phenotype. These results could not be explained by transcriptional changes in the expression levels of *GORK* and *HAK5* transporters in the mature root zone (Figure 5), implying some operational regulation. In the elongation zone; however, WT plants showed strong (four-fold) up-regulation of *HAK5* (Figure 5), while its transcript levels remained unchanged in the NIL(*SKC1*) line. Therefore, the difference in phenotype might be potentially explained by the difference in K^+^ homeostasis in the root apex between NIL(*SKC1*) and WT plants.

A role of K^+^ as a second messenger mediating plant’ adaptive responses to a hostile environment has emerged recently [49,50]. According to this concept, stress-induced K^+^ efflux may represent a ‘metabolic switch’ that allows plants to inhibit energy-consuming anabolic reactions and redirect a large pool of ATP towards defense [50,51]. However, the amount of K^+^ used in signaling should not compromise a plant’s nutritional demand for this element, so the above process occurs in a highly tissue-specific manner and is confined to the root apex [48]. Also, the signaling process is transient, and, once over, the cytosolic K^+^ needs to be restored. Strong upregulation of *HAK5* transcripts (encoding high-affinity K^+^/H^+^ symport) may be an essential part of this process.

Salinity-induced elevation in the cytosolic free Ca^2+^ is an essential component of salt-stress signaling [52]. Amongst other things, such signaling is essential for the activation of the SOS1 Na^+^/H^+^ exchanger that operates in the removal of excessive Na^+^ from the cytosol [5]. The plasma membrane-based NADPH oxidase plays a critical role in salt stress-induced Ca^2+^ elevation. Encoded by *RBOH* genes, together with non-selective Ca^2+^ permeable channels, NADPH oxidase forms the so-called ‘ROS-Ca^2+^ hub” that amplifies Ca^2+^ signals [53] and operates upstream of the plant adaptive networks. In our case, NIL(*SKC1*) plants showed the smallest H_2_O_2_-induced Ca^2+^ uptake, suggesting that higher levels of HKT1;5 expression might have compromised the plant’s ability for ROS and Ca^2+^ signaling. The specific details of this regulation remain a subject for future investigation.

Under our experimental conditions, NIL(*SKC1*) plants showed a more salt-sensitive phenotype compared with WT. This is, in contrast, to the report by Ren et al. [31]. The possible explanation for this inconsistency may be in the strategies and metabolic cost of osmotic adjustment to salt stress. If plants opt for a strategy to exclude Na^+^ from the shoot, they need to rely on *de novo* synthesis of organic osmolytes, which is an energetically expensive process [4] that will entail substantial yield penalties. Thus, if the stress is not too severe (as in our case) and the amount of Na^+^ taken up is reasonable to be dealt with by means of vacuolar sequestration in the shoot, plants may prefer to rely on Na^+^ uptake for osmotic adjustment. This may be the case here. In Ren et al. [31], the authors used a much more severe treatment, for which Na^+^ exclusion from the shoot might be the better option.

Overall, the findings in this work suggest that the results obtained by using mutant plants should be treated with great caution and cannot be taken as mechanistic evidence for the role of a specific gene in question due to the presence of the multiple feedback loops in living organisms. Also, transcriptional profiling and GUS staining may be misleading and provide incomplete information about the operation/function of a specific transporter protein. More emphasis needs to be put, therefore, on *in planta* functional assays.

## 4. Materials and Methods

### 4.1. Plant Material and Growth Conditions

The bulk of experiments was conducted using rice (*Oryza sativa* L. japonica) cv. Koshikari and its near-isogenic line *SKC1* (NIL-*SKC1*). Seeds were kindly provided by Prof D. Xue (Hangzhou Normal University, China). In electrophysiological experiments, these two lines were complemented by *Oshkt1;5* (4A-02764) knock-down (KD) mutant and its *O. sativa* L. *japonica* cv. Dongjin wild type. Seeds of both lines were kindly supplied by Dr. Chang-deok Han from the National Institute of Agricultural Biotechnology, South Korea. Seeds were surface sterilized with 70% ethanol for 1 min and then with 1.5% sodium hypochlorite solution for 30 min and washed with distilled water for five times. Seeds were germinated at 28 °C and 100% relative humidity and kept in a dark room for 48 h for uniform germination. Once the seeds started sprouting, uniformly germinated seeds were transferred into two plastic seedling floats containing Yoshida nutrient solution [54]. Rice seedlings were grown in a greenhouse at 30 °C during the day and 22 °C at night under natural light conditions for 14 days. The uniformly grown seedlings were then exposed to salinity stress by adding 0- and 80-mM salt (NaCl) to Yoshida nutrient solution. Nutrient solutions containing salt were replaced every three days, and the solution pH was adjusted to 5.0 on a daily basis.

### 4.2. Plant Growth Responses and Elemental Content

Rice seedlings were harvested one week after stress onset. Plants were harvested, and root and shoot fresh weights (FW) were measured immediately after the harvest. Plant samples were then oven-dried at 70 °C for three days to a constant weight, and dry weights were measured. For determining leaf Na^+^ and K^+^ content, dry shoot samples were powdered using a Geno Grinder, and ion concentrations were measured using X-ray fluorescence spectrometry Niton XL3t XRF analyzer (Thermo Fisher, Austin, TX, USA). Elemental concentrations were calculated using standard calibration curves optimized for the machine.

### 4.3. Non-Invasive Ion Flux Measurements

Net fluxes of Na^+^, K^+^, and Ca^2+^ were measured from epidermal and stellar root tissues using non-invasive microelectrode ion fluxes measuring the MIFE system (Univ. Tasmania, Australia). Full details of microelectrodes fabrication and calibration and principles of the MIFE ion flux measurements are reported in our previous publications [55,56]. For K^+^ and Ca^2+^ flux measurements, commercial LIX (liquid ion exchangers) were used (catalog number 99,311 for K^+^; 99,310 for Ca^2+^, all from Sigma-Aldrich, St. Louis, MO, USA). For Na^+^ flux, the calixarene-based microelectrodes with superior Na^+^ selectivity were used [57].

For epidermal flux measurements, hydroponically grown five-day-old seedlings were immobilized in a measuring chamber in horizontal position and left for equilibration for 1 h. The bathing medium (BSM solution) consisted of 200 µM NaCl, 100 µM CaCl_2_, and 200 µM KCl (pH 5.5). Ion-selective microelectrodes were positioned next to the root surface, either in elongation (1.2 mm from the tip) or mature (15 mm from the tip) root zones. Steady-state net ion fluxes were measured for 10 min by moving electrodes in a square manner (5 s/5 s cycles) between two positions, 40 and 90 μm above the root surface. After 10 min of recording in control conditions, the treatment was administered by adding either 80 mM NaCl or 10 mM H_2_O_2_ to the chamber, and transient flux responses were recorded for another 30–40 min. Net ion fluxes were then calculated using the MIFEFLUX software assuming a cylindrical diffusion profile [55].

For flux measurements from the xylem parenchyma tissue, root stele was mechanically isolated using the procedure described by Shabala et al. [58]. The isolated stellar segments were allowed to float on a surface of BSM solution for 3–4 h, to avoid any potential confounding effects of mechanical damage during segment isolation. Then the stellar segments were immobilized in the measuring chamber, and transient flux responses from the xylem parenchyma were measured as described above for epidermal cells.

### 4.4. Gene Expression Analysis

Root tissues were harvested from the control and salt-treated plants. The root apex (the first 2–3 mm from the tip) composed from the meristem and elongation (EZ) zone cells was excised with a scalpel, and another 20 mm-long-segment was cut from the remaining mature (MZ) root zone. Samples were immediately frozen in liquid nitrogen. Three independent replicates were collected, each containing pooled samples from six different plants. The total RNA was extracted using the TRIzol reagent (Invitrogen, Carlsbad, CA, USA), and reverse transcription was performed according to the protocol of the SensiFAST cDNA synthesis kit (Bioline, London, UK). *SKC1* transcripts levels were analyzed by amplifying with 25 PCR cycles using real-time quantitative RT-PCR Quantinova Sybr Green Kit in a Rotor-Gene 3000 quantitative PCR instrument (Corbett Research, Mortlake, NSW, Australia). Os*GAPDH* was used as a reference gene.

## Abbreviations

WT	Wild type
NIL(*SKC1*)	Near-isogenic line with a short segment containing *SKC1*
KD	Knock-down
HKT	High-affinity K^+^ Transporter
GORK	Guard cell Outward Rectifying K^+^ channel
FW	Fresh weight
DW	Dry weight
ROS	Reactive Oxygen Species
SOS	Salt Overly Sensitive
QTL	Quantitative Trait Locus
MIFE	Microelectrode Ion Flux Estimation
LIX	Liquid Ion Exchanger
BSM	Basic Salt Medium
EZ	Root elongation zone
MZ	Root mature zone
